# Exploring the mechanism of aloe-emodin in the treatment of liver cancer through network pharmacology and cell experiments

**DOI:** 10.3389/fphar.2023.1238841

**Published:** 2023-10-12

**Authors:** Mingyang Zhu, Qingmin He, Yanan Wang, Liying Duan, Kang Rong, Yingying Wu, Ye Ding, Yang Mi, Xiaoyang Ge, Xiaocui Yang, Yong Yu

**Affiliations:** ^1^ Department of Gastroenterology, The Fifth Affiliated Hospital of Zhengzhou University, Zhengzhou, Henan, China; ^2^ Henan Key Laboratory of Helicobacter Pylori & Microbiota and Gastrointestinal Cancer, Marshall B. J. Medical Research Center of Zhengzhou University, The Fifth Affiliated Hospital of Zhengzhou University, Zhengzhou, Henan, China; ^3^ Department of Gastroenterology, Ankang Central Hospital, Ankang, Shaanxi, China; ^4^ Academy of Medical Science, Zhengzhou University, Zhengzhou, Henan, China

**Keywords:** molecular docking, network pharmacology, aloe-emodin, hepatocellular carcinoma, molecular mechanism, apoptosis

## Abstract

**Objective:** Aloe-emodin (AE) is an anthraquinone compound extracted from the rhizome of the natural plant rhubarb. Initially, it was shown that AE exerts an anti-inflammatory effect. Further studies revealed its antitumor activity against various types of cancer. However, the mechanisms underlying these properties remain unclear. Based on network pharmacology and molecular docking, this study investigated the molecular mechanism of AE in the treatment of hepatocellular carcinoma (HCC), and evaluated its therapeutic effect through *in vitro* experiments.

**Methods:** CTD, Pharmmapper, SuperPred and TargetNet were the databases to obtain potential drug-related targets. DisGenet, GeneCards, OMIM and TTD were used to identify potential disease-related targets. Intersection genes for drugs and diseases were obtained through the Venn diagram. Gene Ontology (GO) and Kyoto Encyclopedia of Genes and Genomes (KEGG) enrichment analyses of intersecting genes were conducted by the website of Bioinformatics. Intersection genes were introduced into STRING to construct a protein-protein interaction network, while the Cytoscape3.9.1 software was used to visualize and analyze the core targets. AutoDock4.2.6 was utilized to achieve molecular docking between drug and core targets. *In vitro* experiments investigated the therapeutic effects and related mechanisms of AE.

**Results:** 63 overlapped genes were obtained and GO analysis generated 3,646 entries by these 63 intersecting genes. KEGG analysis mainly involved apoptosis, proteoglycans in cancer, TNF signaling pathway, TP53 signaling pathway, PI3K-AKT signaling pathway, etc. AKT1, EGFR, ESR1, TP53, and SRC have been identified as core targets because the binding energies of them between aloe-emodin were less than -5 kcal/Mol.The mRNA and protein expression, prognosis, mutation status, and immune infiltration related to core targets were further revealed. The involvement of AKT1 and EGFR, as well as the key target of the PI3K-AKT signaling pathway, indicated the importance of this signaling pathway in the treatment of HCC using AE. The results of the Cell Counting Kit-8 assay and flow analysis demonstrated the therapeutic effect of AE. The downregulation of EGFR, PI3KR1, AKT1, and BCL2 in mRNA expression and PI3KR1, AKT,p-AKT in protein expression confirmed our hypothesis.

**Conclusion:** Based on network pharmacology and molecular docking, our study initially showed that AE exerted a therapeutic effect on HCC by modulating multiple signaling pathways. Various analyses confirmed the antiproliferative activity and pro-apoptotic effect of AE on HCC through the PI3K-AKT signaling pathway. This study revealed the therapeutic mechanism of AE in the treatment of HCC through a novel approach, providing a theoretical basis for the clinical application of AE.

## 1 Introduction

Liver cancer is one of the leading causes of cancer-related death worldwide, with a gradually increasing incidence annually (Siegel et al., 2019). In 2018, based on data from 185 countries and territories, liver cancer ranked sixth and third in incidence and mortality, respectively (Sung et al., 2021). Hepatitis viruses, smoking, obesity, diabetes, and certain dietary habits are risk factors of liver cancer (Yu, 1995). Despite a clear etiology, the pathogenesis and molecular mechanisms of liver cancer remain unclear. Liver cancer consists of two main histopathological types, namely, hepatocellular carcinoma (HCC) and intrahepatic cholangiocarcinoma. HCC accounts for 83.9%–92.3% of all liver cancer cases in China. Approximately 1 million patients are diagnosed with HCC worldwide on an annual basis. The prognosis for patients with liver cancer is extremely poor. Surgery is only appropriate in early-stage disease; consequently, surgical resection is possible in <15% of patients. Treatment options for patients with advanced cancer are relatively limited. Transarterial chemoembolization is a typical surgical option for such patients, increasing 2-year survival by 23% *versus* that of patients with intermediate-stage HCC who receive conservative treatment. Sorafenib, a molecularly targeted agent with good efficacy against liver cancer, is another treatment option. This drug can inhibit the proliferation of cancer cells, and is indicated for the treatment of patients with advanced liver cancer. Nevertheless, an increasing number of patients develop tolerance to chemotherapeutic drugs, and only a minority of patients with HCC benefit from continued chemotherapy (El-Serag et al., 2008). In addition, drug toxicity and/or ineffectiveness after long-term use cannot be ignored. Current radiofrequency ablation and chemotherapy regimens cannot completely cure this catastrophic disease. Thus, further research studies are warranted to discover a more effective treatment for liver cancer.

Traditional Chinese herbal medicines have attracted the attention of researchers owing to their low rates of side effects, low toxicity, and cost-effectiveness compared with conventional chemotherapeutic drugs. Aloe-emodin (AE) is an anthraquinone compound extracted from the roots, stems, and leaves of aloe vera, rhubarb, cassia, and other plants. It exerts a broad range of antibacterial, antiviral, and anti-inflammatory effects. Previous study has demonstrated that AE could restrain the lipopolysaccharide-induced production of proinflammatory cytokines in RAW264.7 macrophages via downregulation of nuclear factor-κB (NF-κB), mitogen-activated protein kinase (MAPK), and phosphatidylinositol 3 kinase (PI3K) (Hu et al., 2014). AE also inhibits the formation of *Staphylococcus aureus* by reducing the production of extracellular proteins (Xiang et al., 2017). Studies on Japanese encephalitis virus and enterovirus 71 revealed the antiviral activity of AE (Lin et al., 2008). Further research on AE discovered its anticancer effects. At present, AE has been utilized for the treatment of lung cancer (Wu et al., 2017a), gastric cancer (Guo et al., 2008), colon cancer (Suboj et al., 2012), liver cancer (Lu et al., 2007), and other types of cancer. However, the mechanism underlying the effects of AE in HCC remains unknown.

Network pharmacology is an emerging method which not only combines network biology and poly-pharmacology to validate drug-actionable targets through computational software, but also explores potential mechanisms of drug therapeutic actions (Hopkins, 2008). Network pharmacology also provides a way of thinking about drug discovery while being able to understand the side effects and toxicity of drugs. This method has completely altered the approach to the definition, diagnosis, and treatment of diseases (Nogales et al., 2022). The 3D modeling of the drug and protein receptor can be constructed by computer software, which can screen the optimal sites on the protein receptor for amino acid-ligand docking. In turn, these genetic proteins are inextricably linked to disease, and thus a web of relationships between drugs and disease has been established. Furthermore, the strength of the association between pivotal genes and drugs can be confirmed by molecular docking. Therefore, in this study, we used network pharmacology and molecular docking to explore the specific molecular mechanisms of AE for the treatment of HCC and validate its antitumor effects through cell experiments.

## 2 Materials and methods

### 2.1 Network pharmacology

#### 2.1.1 Target prediction for AE

**FIGURE 1:**
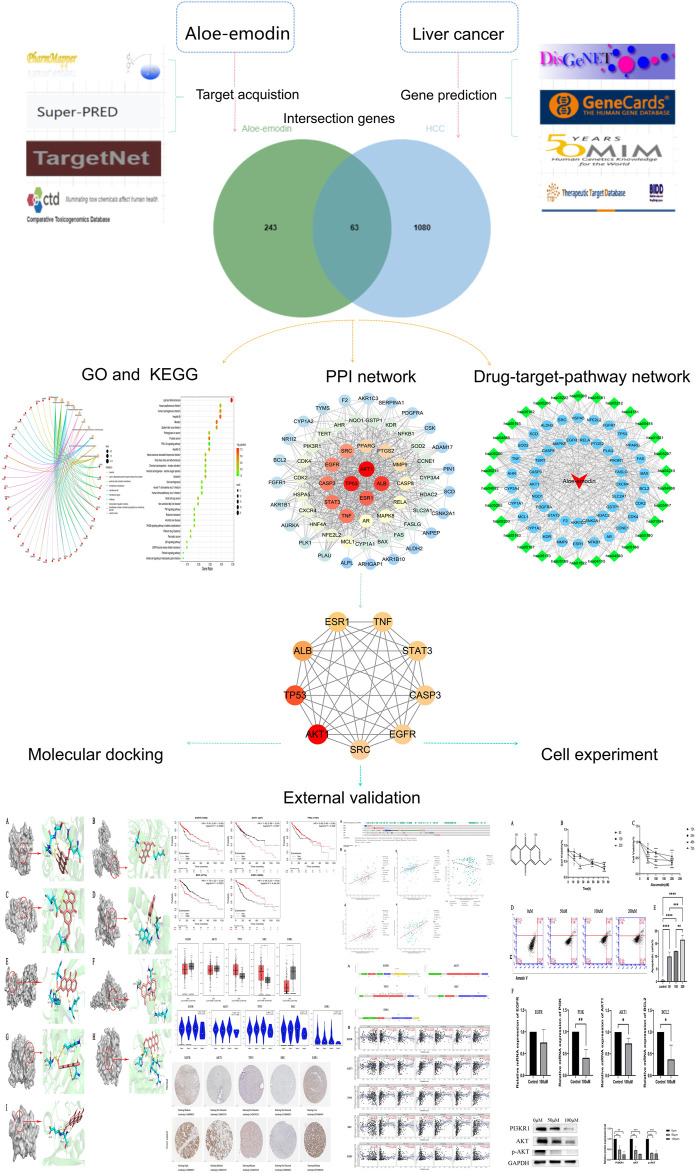
A detailed graphic summary of network-pharmacology. (Network pharmacology process, molecular docking, external validation of core targets and experimental verification *in vitro*).

The workflow of this analysis is shown in Figure 1. Isomeric SMILES of AE was obtained from PubChem, the world’s largest chemical information base where we could get chemical structures, chemical properties, biological activities of small molecules gratis (Kim et al., 2023). The Comparative Toxicogenomics Database (CTD), Pharmmapper, Superpred, and TargetNet databases were screened to identify potential target genes. These four databases were designed to predict drug-related genes (Nickel et al., 2014; Yao et al., 2016; Wang et al., 2017; Davis et al., 2023). “Human Species” was set as a requirement for this analysis. Following the removal of duplicates, the selected targets were standardized using the UniProt database. The genes screened from these four databases under certain conditions were the genes associated with the therapeutic effects of aloe-emodin. Aloe-emodin may exert its therapeutic effects towards diseases through interactions between these genes.

#### 2.1.2 Identification of potential targets in HCC and intersection genes

Liver cancer-related genes were recognized using four genetic databases, namely, DisGenet (Pinero et al., 2021), GeneCards (Stelzer et al., 2016), Online Mendelian Inheritance in Man (OMIM) (Amberger et al., 2015), and Therapeutic Target Database (Zhou et al., 2022). Based on the rankings, we searched for mutated genes that are more likely to be involved in the development and progression of HCC, and used UniProt to normalize these disease-associated genes. The drug and disease genes were subsequently mapped, and the mapping results were imported into the jvenn website (Bardou et al., 2014) to obtain intersection genes. Therapeutic effects of aloe-emodin on hepatocellular carcinoma were likely to be mediated by modulation of these intersecting genes.

#### 2.1.3 Gene ontology (GO) and kyoto encyclopedia of genes and genomes (KEGG) enrichment analysis

GO is a biological system used for studying the effects of individual genes on an organism at different biological levels. KEGG provides evidence indicating the potential involvement of individual genes in biological signaling pathways (Kanehisa et al., 2022). GO and KEGG online analysis module in the Bioinformatics website were used to conduct the enrichment analysis. When the list of intersecting genes were imported, selected the species as “human”, ran the program until the results of the enrichment analysis and related images were exported.

#### 2.1.4 Drug-target-pathway network construction

The drug-target-pathway network was established by introducing intersection genes and KEGG signaling pathway items into Cytoscape 3.9.1. Cytoscape is a platform that integrates complex network structures with data and presents them in graphical form (Shannon et al., 2003). The cellular nodes represent AE, intersection genes, or pathways, while the connecting lines between different nodes represent genes involved in different pathways.

#### 2.1.5 Protein-protein interaction (PPI) network construction and visualization

PPI is a method utilized to study the mechanism through which proteins function harmoniously in cells (Ding and Kihara, 2019). Using the STRING database (version 11.5), we constructed the PPI network with the species limited to “*Homo sapiens*” and a medium confidence score of 0.4 to ensure more protein-protein information would be included. The PPI network was updated after removing disconnected nodes. The tab-separated values (tsv) format file was downloaded and imported into Cytoscape 3.9.1 for subsequent visualization. The strength of interactions between proteins in Cytoscape would be calculated in order to screen for core targets.

#### 2.1.6 Screening of core targets

Using the “CytoNCA” plug-in in Cytoscape 3.9.1, we determined the core targets according to their degree values. “CytoNCA” was a plug-in for calculating the strength of interactions between proteins, some proteins with higher interaction strengths with other proteins could be screened out as core targets (Tang et al., 2015). Several methods for calculating the strength of protein associations were included in this plug-in and one of them-degree has been chosen as our method for screening core targets. The intersecting genes with degree values more than two-fold higher than the median were selected as core targets. The genes were imported into the plug-in of MCODE for hub gene visualization.

#### 2.1.7 Molecular docking between AE and hub genes

We sought to further understand the relationship between candidate proteins and AE, as well as their mechanism of action. Therefore, molecular docking was performed to determine the strength of the interaction between receptors and ligands. The SDF (Structural Data File) of AE was downloaded from PubChem, and the Protein Data Bank (PDB) database was used to obtain the SDF format file of the original ligand. Moreover, the pdb format files of receptor proteins were obtained from the PDB database (Table 1). The SDF format files of AE and the original ligand were transformed into mol2 format via OpenBabel-3.1.1 (O'Boyle et al., 2011). The receptor proteins were introduced into PyMOL 2.5 (Seeliger and de Groot, 2010) for dehydrating and deligand. Thereafter, we modified the receptor proteins with hydrogenation in AutoDockTools 1.5.6 (Morris et al., 2009) and extracted the files in pdbqt format for further operation. Setting torsion for ligand and outputting ligand as pdbqt format. The qdbqt format files of receptor proteins and ligands were re-imported into AutoDockTools for analysis using AutoGrid. In AutoGrid, all branches of the receptor proteins were entirely covered by GridBox. The parameters of the GridBox were recorded (Table 2). Next, AutoGrid was used to generate files in gpf format. After setting the parameters of docking, the gpf format file was imported into AutoDock, and the docking process was initiated. At the end of the process, the binding energy of each receptor protein and ligand was recorded and saved in pdbqt format. The results of molecular docking were visualized though PyMOL. All websites used in this analysis are shown in Table 3.

**TABLE 1:** Details of the protein targets in the PDB database.

Targets	PDB ID	Method	Resolution	R-Value free	R-Value work	R-Value observed
AKT1	7NH5	X-RAY DIFFRACTION	1.90 Å	0.228	0.200	0.201
TP53	8DC6	X-RAY DIFFRACTION	1.60 Å	0.202	0.181	0.182
ALB	6YG9	X-RAY DIFFRACTION	1.89A	0.313	0.239	0.243
ESR1	5FQV	X-RAY DIFFRACTION	1.74 Å	0.234	0.193	0.195
TNF	1EXT	X-RAY DIFFRACTION	1.85 Å	0.243	0.203	0.203
STAT3	6NUQ	X-RAY DIFFRACTION	3.15 Å	0.260	0.233	0.234
CASP3	1NMS	X-RAY DIFFRACTION	1.70 Å	0.183	0.151	0.153
EGFR	3POZ	X-RAY DIFFRACTION	1.50 Å	0.243	0.219	0.219
SRC	1O43	X-RAY DIFFRACTION	1.50 Å	-	0.1986	0.196

**TABLE 2:** Grid docking parameters in molecular docking.

Targets	PDB ID	Spacing (angstrom)	Center grid box		
			X center	Y center	Z center
AKT1	7NH5	0.642	13.893	−11.833	−15.603
TP53	8DC6	0.975	39.591	−6.574	−12.787
ALB	6YG9	0.875	28.936	0.178	18.993
ESR1	5FQV	0.531	12.653	33.952	69.296
TNF	1EXT	0.847	−0.382	32.548	−4.553
STAT3	6NUQ	1.000	−2.205	19.287	24.548
CASP3	1NMS	0.703	11.911	0.021	14.823
EGFR	3POZ	0.636	19.556	24.903	15.074
SRC	1O43	0.458	10.597	19.284	19.914

**TABLE 3:** Basic information of the database used for the screening of aloe-emodin in the treatment of liver cancer.

Database	Website
PubChem	https://pubchem.ncbi.nlm.nih.gov
CTD	https://ctdbase.org
PharmMapper	http://lilab-ecust.cn/pharmmapper/index.html
SuperPred	https://prediction.charite.de
TargetNet	http://targetnet.scbdd.com/
Uniprot	https://www.uniprot.org/
DisGeNET	https://www.disgenet.org/
GeneCards	https://www.genecards.org/
OMIM	https://www.omim.org/
TTD	https://db.idrblab.net/ttd/
jvenn	https://jvenn.toulouse.inra.fr/app/index.html
Cytoscape	https://cytoscape.org/
STRING	https://cn.string-db.org/
PDB	https://www.rcsb.org/
GEPIA	http://gepia.cancer-pku.cn/
HPA	https://www.proteinatlas.org/
Kaplan-Meier plotter	https://kmplot.com/
cBioPortal	https://www.cbioportal.org/
TIMER	https://cistrome.shinyapps.io/timer/

#### 2.1.8 External validation of core targets

##### 2.1.8.1 mRNA, protein expression levels and survival analysis of core targets

We analyzed the transcript and protein levels of five core targets in HCC cells *versus* normal hepatocytes using the Gene Expression Profiling Interactive Analysis (GEPIA) database (Tang et al., 2017) and the Human Protein Atlas database (Uhlen et al., 2015). Pathological stage analysis of core targets in HCC was performed to verify the change in mRNA expression at different stages of the disease. The correlation between the mRNA and protein levels of core targets was also analyzed using cBioPortal database (Cerami et al., 2012). Next, we explored the role of core target mutations in the prognosis of patients with HCC. Thus, we used the Kaplan-Meier plotter database (Gyorffy, 2023) to analyze the odds of survival in HCC patients with inconsistent core target expression.

##### 2.1.8.2 Immune cell infiltration and genetic alteration of core targets

When cancer occurs, it is especially prominent to know about gene mutations and changes in the immune system (Abbott and Ustoyev, 2019; Paul et al., 2019). The Tumor Immune Estimation Resource (TIMER) database (Li et al., 2017) was used to observe the relevance between the expression of hub genes and immune cell infiltration in HCC. The abundance of six immune infiltrates (i.e., B cells, CD4^+^ T cells, CD8^+^ T cells, neutrophils, macrophages, and dendritic cells) was calculated using TIMER. And genetic alterations and genetic mutation sites of core targets were detected using cBioPortal database.

### 2.2 Biological testing

#### 2.2.1 Test compound and cell culture

AE (100 mg) was purchased from TCI Chemicals (Shanghai, China), dissolved in dimethyl sulfoxide (60 mg: 1 mL), and stored at −20°C. The final concentrations of AE used in different experiments were reached with Dulbecco’s modified Eagle’s medium (DMEM) (Sigma Chemical). HepG2 cells (Institute of Biochemistry and Cell Biology, CAS; SCSP-526,Shanghai, China) were maintained in DMEM supplemented with 10% fetal bovine serum (Gibco), 1% penicillin and streptomycin (100 U/mL), 1% glutamax, and 1% sodium pyruvate 100 mM solution at 37°C in a humidified incubator with 5% CO_2_.

#### 2.2.2 Cell viability assay

Cell Counting Kit-8 (CCK8; Beyotime, Shanghai, China) assay was used to analyze cell viability according to the instructions provided by the manufacturer. Cells were seeded into 96-well microplates (3 × 10^3^cells/well in 100 μL of medium) and cultured. AE (0, 50, 100, and 200 μM) was used to treat HepG2 cells cells for 0, 12, 24, 48, 72 h. Subsequently, CCK8 reagent (10 μL) was added to each well containing DMEM (90 μL). CCK8 and DMEM were also added to one well not containing cells (blank controls). Thereafter, a microplate reader was used to measure the optical density (OD) at 415 nm wavelength. Percentage viability was calculated using the following formula: % Viability = (OD of treated cells − OD of blank control)/(OD of negative control − OD of blank control) ×100.

#### 2.2.3 Flow cytometry for the detection of cell apoptosis

The Annexin V-fluorescein isothiocyanate/propidium iodide (Annexin V-FITC/PI) Apoptosis detection Kit (Meilunbio, Shanghai, China) was used to verify the effect of AE on HCC cell apoptosis. Briefly, HepG2 cells were seeded into six-well plates (2 × 10^5^cells/well) and cultured overnight. Following adhesion, the cells were exposed to different concentrations (0, 50, 100, and 200 μM) of AE for 24 h. Subsequently, the cells were collected and incubated with Annexin V-FITC/PI at room temperature in the dark for 15 min according to the user manual. The proportion of apoptotic cells was determined using BDAccuri™ C6Plus flow cytometry (Biosciences, United States).

#### 2.2.4 mRNA expression of core targets in PI3K-AKT pathway

We also evaluated the effect of AE on the transcription of core genes in the PI3K-AKT signaling pathway. Cells were seeded into six-well plates (2 × 10^5^cells/well in 1 mL of DMEM medium) and cultured. Previous studies have shown that AE exerted its greatest inhibitory effect on HCC at the concentration of 100 μM(Jeon et al., 2012). Therefore, when the cells reached their logarithmic growth phase, 100 μM AE was added to the wells. After 24 h of intervention, the cells were collected, and TRIzol reagent (1 mL) was added to extract RNA according to the instructions provided by the manufacturer. The concentration of extracted RNA was measured using a BioDrop spectrophotometer (Biochrom Ltd., United Kingdom). Subsequently, the reverse transcription system (10 μL) was constructed using a reverse transcription kit (Bio-Toyobo, Japan). PowerCycler Gradient polymerase chain reaction (PCR) (Analytik Jena, Germany) was used to run the transcription reaction based on the following parameters: 37°C for 15 min, 98°C for 5 min, and 4°C for 60 min. After transforming RNA into cDNA, the ChamQ Universal SYBR qPCR Master Mix (Vazyme, Nanjing, China) was utilized to perform quantitative PCR. The LightCycler^®^480 II (Roche, Switzerland) was used to conduct the DNA amplification.

#### 2.2.5 Western blot analysis of core targets in PI3K-AKT pathway

HepG2 cells were spread evenly in 6-well plates at 2 × 10^5^ per well, and after the cells were adhered to the wall, 50 μM and 100 μM of aloe-emodin was used to intervene with for 24 h, and then the cells were lysed with high-strength RIPA lysing solution (1%Triton X-100, 1% sodium deoxycholate, 0.1% SDS, 1%protease inhibitor and 1%phosphatase inhibitor) at 150 ul per well for 20 min. The lysed cells were subsequently transferred to 1.5 mL centrifuge tubes, followed centrifugation (14,000 x g) at 4°C for 10 min. Then the centrifuged supernatant was extracted and mixed with 20% loading buffer for Agarose Gel Electrophoresis. Protein samples were then separated on the 10% PAGE gel and transferred to a nitrocellulose membrane. The membrane was incubated with a blocking solution of 5% skim milk powder and Tris-buffered saline with Tween-20 (TBST) for 2 hours at room temperature to block non-specific antibody binding, and then washed three times with TBST for 10 min. Then the membrane was incubated overnight at 4°C using primary antibodies with dilutions of 1:5000. PI3KR1 antibody (60225-1-Ig), AKT1 antibody (30203-2-Ig),p-AKT1 antibody (66444-1-Ig) and GAPDH antibody (60004-1-Ig) were purchased from Proteintech Group (Chicago, United States). The primary antibody was washed 3 times with TBST at the end of the incubation, followed by incubation with the secondary antibody conjugated to horseradish peroxidase (Abways Technology, Shanghai,China) with dilution of 1:10000 for 1 h at room temperature. Then the protein bands was added with ECL luminescence reagent (Meilunbio, Shanghai,China) for visualization by ChemiDoc XRS + System (Bio-Rad,California,United States). The Western blot results were quantified using Fiji (2.14.0) software (Schindelin et al., 2012).

#### 2.2.6 Statistical analysis

Statistical significance between two or multiple groups was assessed using Student’s *t*-test and one-way analysis of variance (ANOVA), respectively. The mean ± standard deviation was used to plot the data. *p*-values < 0.05 indicate statistically significant differences. The analysis was performed using the GraphPad Prism software version 9.0 (GraphPad Software Inc., San Diego, CA, United States of America) (Berkman et al., 2019).

## 3 Results

### 3.1 Network pharmacology based-analysis

#### 3.1.1 Identification of targets and intersection genes

**FIGURE 2:**
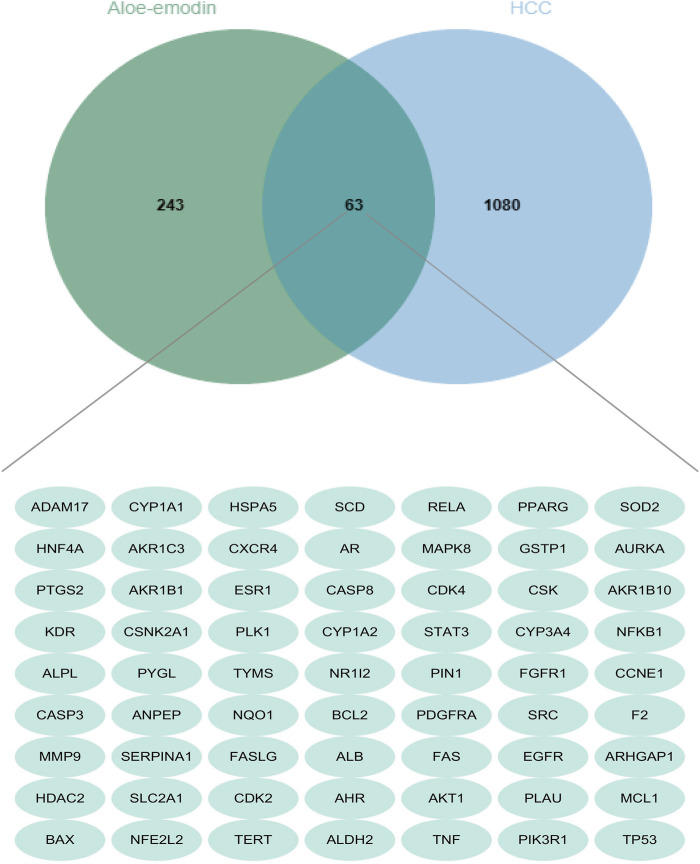
Venn diagram displays the intersection genes of aloe-emodin and hepatocellular carcinoma. (The green circle represents a total of 306 aloe-emodin-related genes screened from several databases, the blue portion represents a total of 1,143 liver cancer-related genes screened from databases, and the cross section in the middle represents the 63 intersecting genes).

A total of 127 genes were identified from Pharmmapper according to a norm fit score ≥0.4. In addition, 109 and 52 genes were selected from SuperPred and TargetNet, respectively, based on a probability ≥0.5. Moreover, 47 genes were selected from the Comparative Toxicogenomics Database. Following the removal of duplicates, a total of 306 drug-related genes were obtained.1143 disease-related genes were identified from Disgenet, Genecards, OMIM, and Therapeutic Target Database. As shown in the Venn diagram (Figure 2), 63 genes were matched. Some intersecting targets could be present on pathways associated with hepatocellular carcinoma development and play important regulatory roles. Therefore, we next performed KEGG enrichment analysis to understand the cancer pathways enriched by these intersecting genes, which were the regulatory mechanisms of aloe-emodin on hepatocellular carcinoma.

#### 3.1.2 GO or KEGG enrichment analysis

**FIGURE 3:**
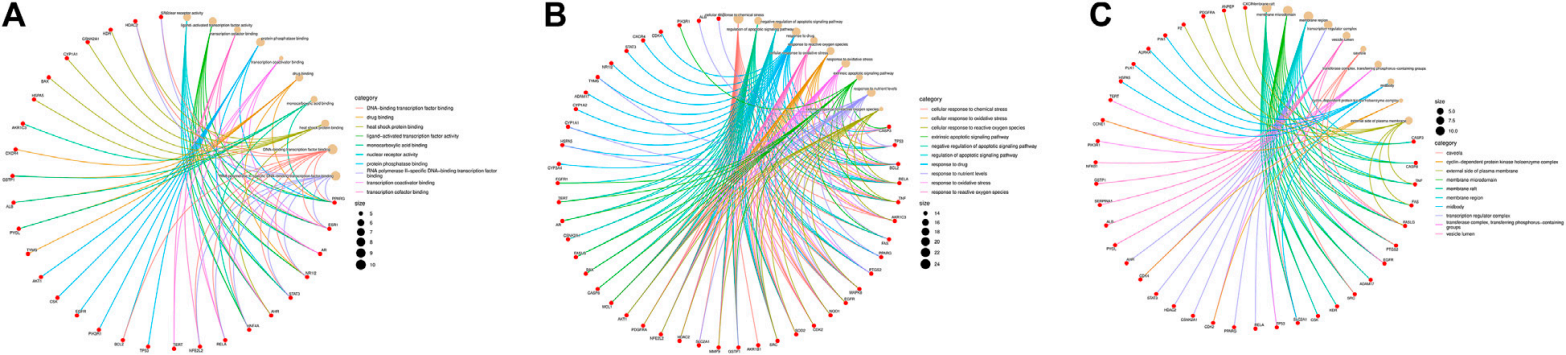
Pie charts of GO functional enrichment analysis of aloe-emodin in hepatocellular carcinoma. [Red represents genes and brown represents biological processes. The connecting lines between genes and biology represent the genes involved in each biological process. The size of the brown circle represents the number of genes involved in the biological process. **(A)** Biological process of aloe-emodin in HCC. **(B)** Cellular component of aloe-emodin in HCC. **(C)** Molecular function of aloe-emodin in HCC)].

**FIGURE 4:**
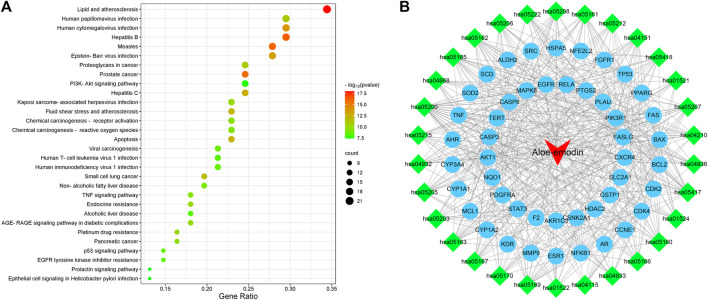
Bubble plot of KEGG enrichment analysis. [**(A)** KEGG pathway enrichment analysis of aloe-emodin in hepatocellular carcinoma. The redder color of the circle represents a smaller *p*-value, indicating a regulatory pathway that these intersecting genes are more likely to be involved in. The size of the circle represents the number of genes involved in regulating this pathway **(B)**. Drug-target-pathway network diagram. The blue circles represent targets, the green diamonds are pathways, and the red triangles is aloe-emodin. This figure suggests that aloe-emodin may exert its therapeutic effects on hepatocellular carcinoma by modulating multi-targets and multi-pathways].

These data were input into the Bioinformatics website for further GO and KEGG enrichment analyses. GO analysis generated 3,646 entries which were classified in biological process (n = 3,116), cellular component (n = 194), and molecular function (n = 336). The top 10 entries of each type were filtered based on the *p*-value, and the genes contained in each type are shown (Figure 3). Red nodes represent genes; brown nodes represent diverse categories of GO; and the size of brown nodes represents numbers of genes. The genes corresponding to each category were connected with lines of different colors. The biological process ontology mainly included cellular response to chemical stress, negative regulation of apoptotic signaling pathway, regulation of apoptotic signaling pathway, response to drug, etc. The cellular component ontology was constituted by membrane raft, membrane microdomain, membrane region, transcription regulator complex, etc. The molecular function ontology included nuclear receptor activity, ligand-activated transcription factor activity, transcription co-factor binding, protein phosphatase binding, transcription coactivator binding, etc. KEGG enrichment analysis yielded 227 entries; the first 30 KEGG signaling pathways were identified according to the *p-*value (Figure 4A). The main cancer pathways which were enriched included apoptosis, proteoglycans in cancer, the tumor necrosis factor (TNF) signaling pathway, tumor protein p53 (TP53) signaling pathway, PI3K-AKT signaling pathway, and prolactin signaling pathway. The genes involved in each signaling pathway were visualized using Cytoscape, indicating that the activation of multiple signals might play a role in the regulation of HCC after treatment with AE (Figure 4B). Thus, aloe-emodin may exert its therapeutic effects on hepatocellular carcinoma by modulating these pathways enriched by these 63 intersecting genes. Aloe-emodin inhibited the proliferation of liver cancer cells by modulating one or some of the targets on these cancer pathways.

#### 3.1.3 PPI network construction and selection of core targets

**FIGURE 5:**
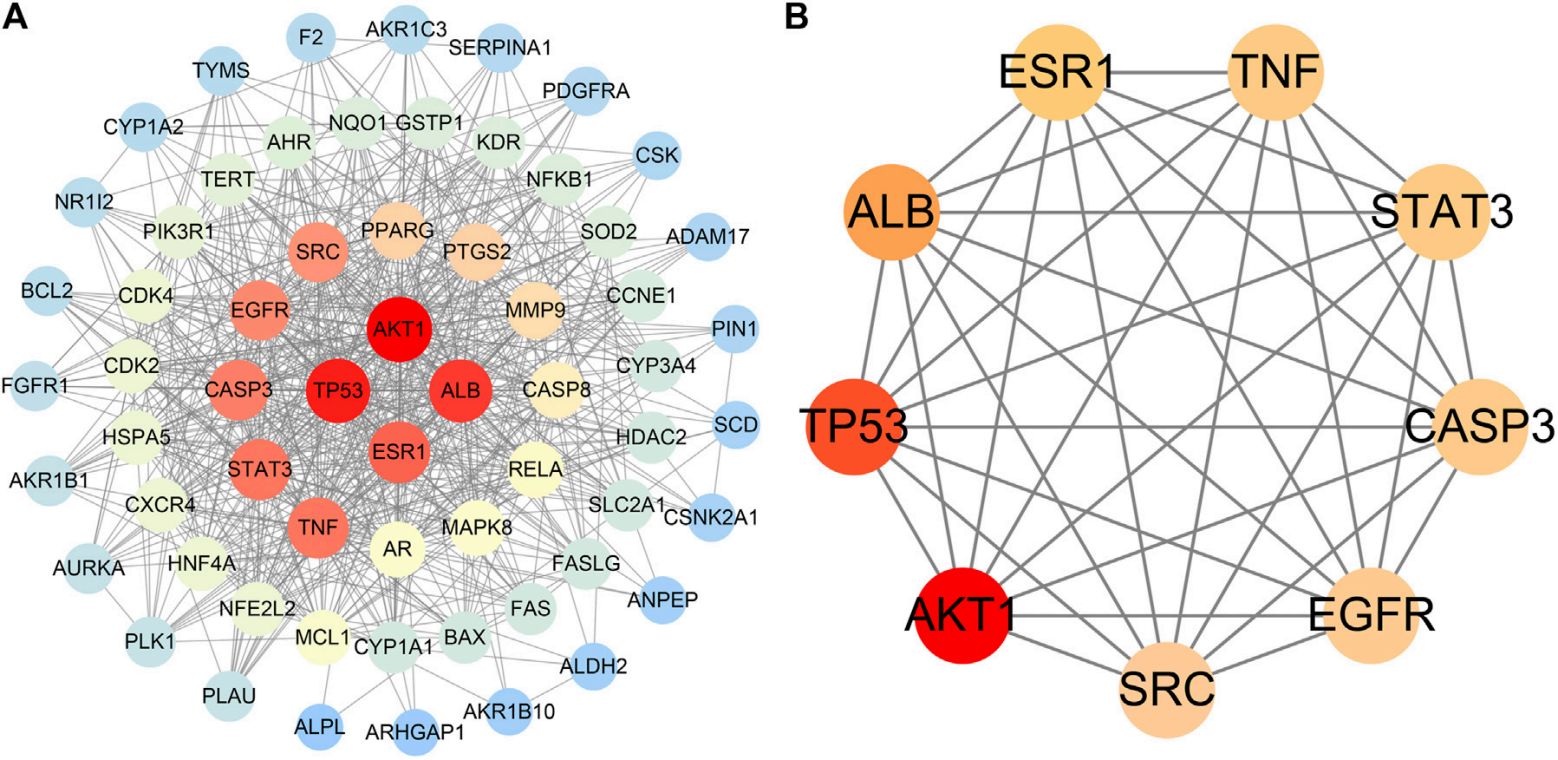
PPI network diagram. [**(A)** PPI network of potential targets for aloe-emodin therapy of hepatocellular carcinoma. **(B)** Core targets selected by degree value. The color shade represents the size of the degree value].

The PPI network included a total of 63 nodes and 628 edges. The average node degree and PPI enrichment *p*-value remained 19.9 and <1.0e-16, respectively, suggesting that these proteins were at least biologically related. Thereafter, Cytoscape was used for PPI visualization (Figure 5A). We used“Degree”algorithms in the plug-in “CytoNCA” to calculate the levels of each protein. Degree represents the number of lines in the protein node, and the more lines a protein has, the more important role it has in protein interactions. (Table 4). The size of the circle in the Figure represents the levels; moreover, the color intensity of the circle increase in parallel with the levels of expression (Figure 5B). The results showed that AKT serine/threonine kinase 1 (AKT1), TP53, albumin (ALB), estrogen receptor 1 (ESR1), TNF, signal transducer and activator of transcription 3 (STAT3), caspase 3 (CASP3), epidermal growth factor receptor (EGFR), and SRC proto-oncogene (SRC) may be the key factors in the treatment of HCC using AE.

**TABLE 4:** 10 Hub genes identified using degree value by CytoNCA in Cytoscape.

Target name	Full name	Degree
AKT1	AKT serine/threonine kinase 1	106
TP53	Tumor protein p53	100
ALB	Albumin	94
ESR1	Estrogen receptor 1	86
TNF	Tumor necrosis factor	82
STAT3	Signal transducer and activator of transcription 3	82
CASP3	Caspase 3	80
EGFR	Epidermal growth factor receptor	78
SRC	SRC proto-oncogene	76

#### 3.1.4 Molecular docking validation of AE and core targets

**FIGURE 6:**
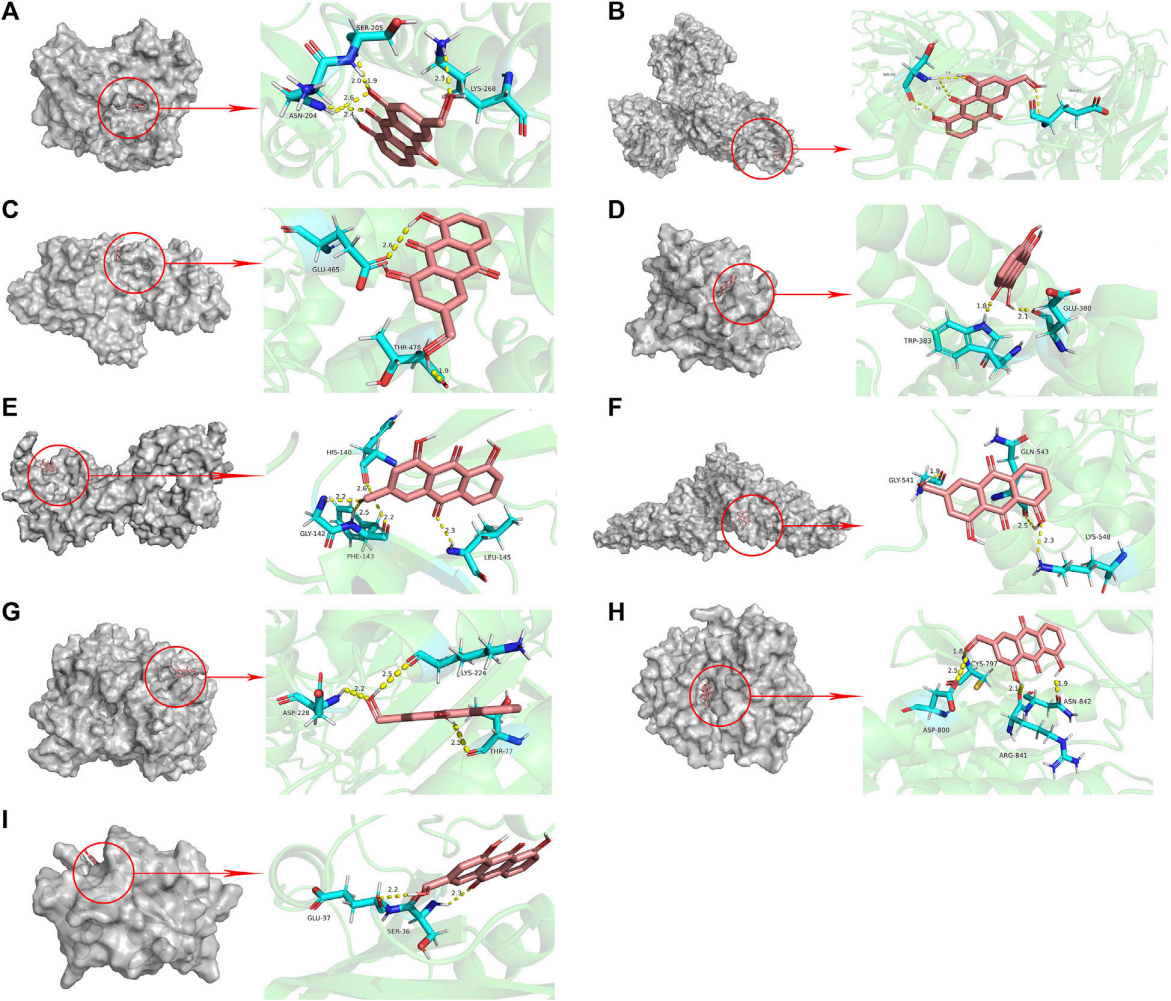
Molecular docking pattern of aloe-emodin and core target protein. [The gray portion on the left represents the surface location of aloe-emodin on the protein receptor, and the right represents the name of the specific amino acid that aloe-emodin binds to each protein, and the length and number of hydrogen bonds **(A)** Aloe-emodin-AKT1, **(B)** Aloe-emodin-TP53, **(C)** Aloe-emodin-ALB, **(D)** Aloe-emodin-ESR1, **(E)** Aloe-emodin-TNF, **(F)** Aloe-emodin-STAT3, **(G)** Aloe-emodin-CASP3, **(H)** Aloe-emodin-EGFR, **(I)** Aloe-emodin-SRC)].

**FIGURE 7:**
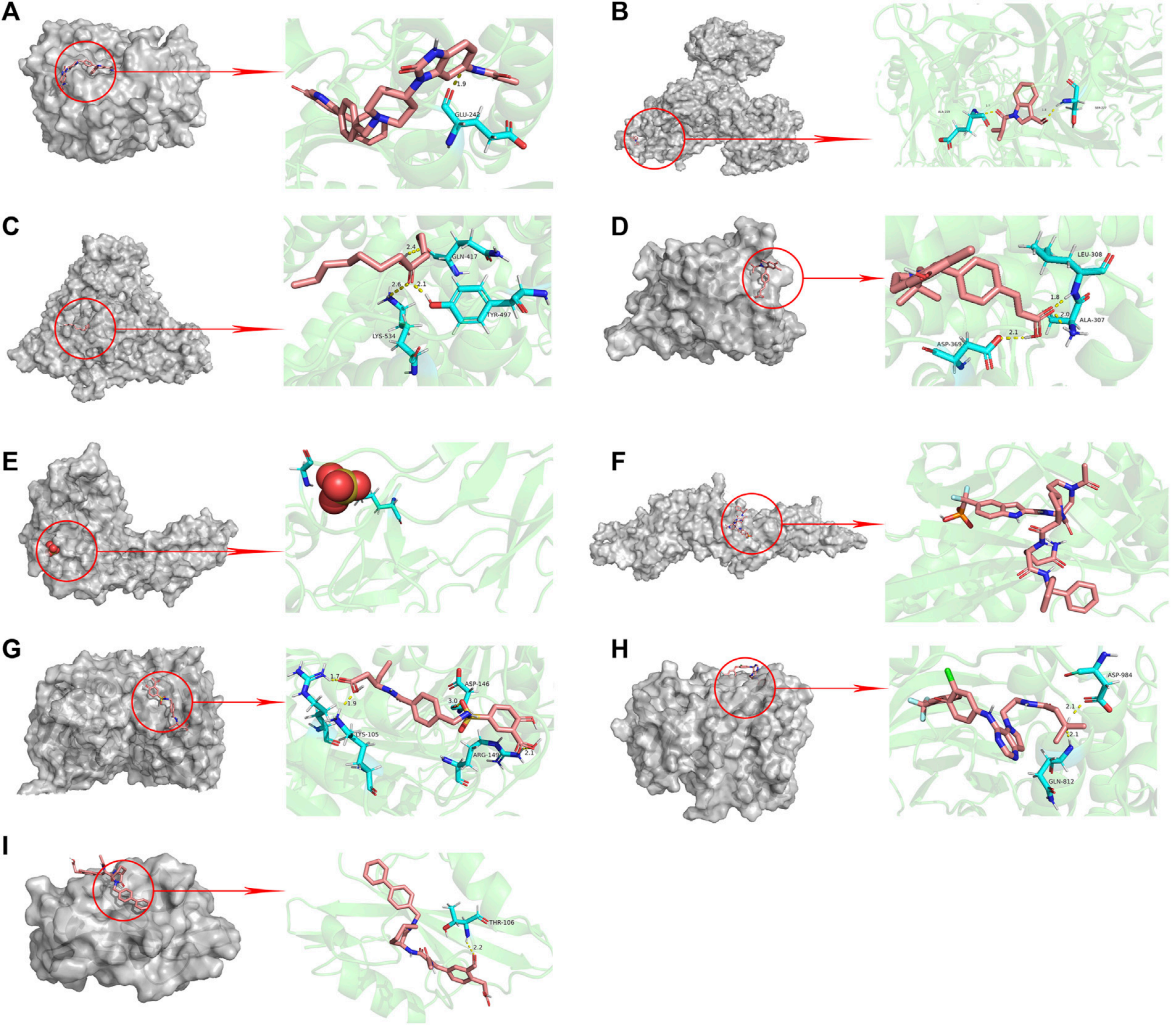
Molecular docking pattern of original ligand and core target protein. [The gray portion on the left represents the surface location of their original ligands on the protein receptor, and the right represents the name of the specific amino acid that their original ligands bind to each protein, and the length and number of hydrogen bonds **(A)** UC8-AKT1, **(B)** R4F-TP53, **(C)** MYR-ALB, **(D)** VQI-ESR1, **(E)** SO4-TNF, **(F)** KQV-STAT3, **(G)** 161-CASP3, **(H)** 03P-EGFR, **(I)** 821-SRC)].

**FIGURE 8:**
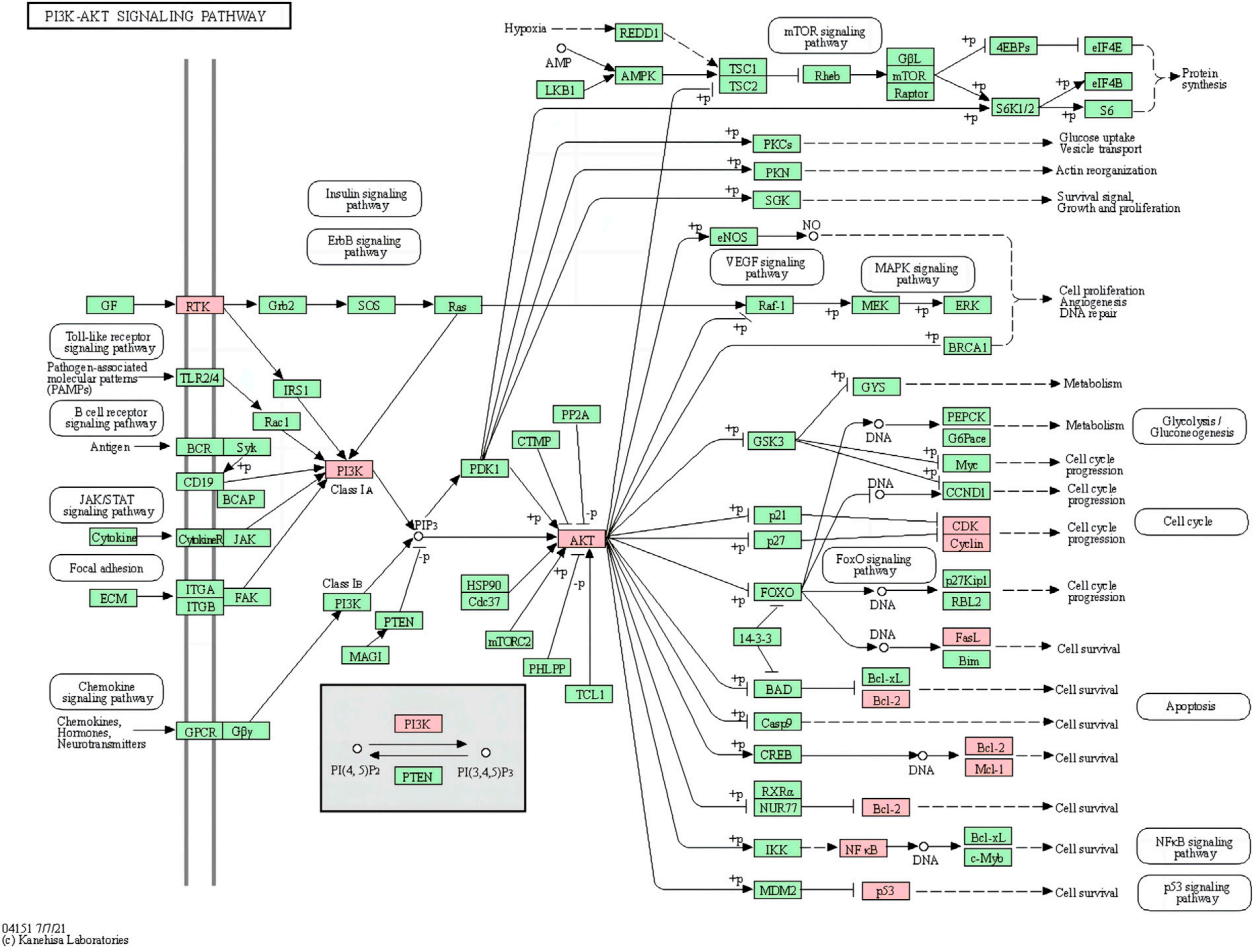
PI3K-AKT signaling pathway (pink marks represent potential targets for aloe-emodin intervention).

The results of molecular docking showed that the values of the docking energy of AE and candidate proteins were <0 (Table 5), indicating that AE could spontaneously bind to the amino acids of target proteins without external assistance (He et al., 2023). Among them, AKT1, TP53, ALB, ESR1, STAT3, CASP3, and EGFR had higher binding energy compared with their original ligands. SRC had similar binding energy to that of its original ligand. AKT1, TP53, ESR1, SRC, and EGFR had energy <−5 kcal/Mol, demonstrating that these five core targets of AE play an important role in the treatment of HCC(Li et al., 2021; Liu et al., 2021; Li T. et al., 2022). The docking results were visualized through PyMOL (Figures 6, 7). Dysfunction of a variety of signaling pathways is associated with the occurrence and progression of cancer. Previous studies have shown that AE could exert its therapeutic effect on HCC by upregulating the expression of TP53. The PI3K-AKT signaling pathway is a classical dysregulated pathway involved in the pathogenesis of HCC, and half of HCC patients present with mutations in PI3K-AKT. Consistently, key targets of this signaling pathway were also detected through our KEGG enrichment analysis (Figure 8). A previous study demonstrated AE could suppress the proliferation of lung cancer cells via the PI3K-AKT pathway (Wu et al., 2017b). Therefore, we hypothesized that AE might suppress the proliferation of HCC cells and promote apoptosis via the PI3K-AKT signaling pathway. External validation of core targets and cell experiments were conducted to confirm our hypothesis.

**TABLE 5:** Basic information on the molecular docking of aloe-emodin and target proteins.

Ligand	Targets	Residues	Hydrogen bond length	Bindig energy (kcal/Mol)
Aloe-emodin	AKT1	ASN-204; SER-205; LYS-268	2.6,2.4; 2.0,1.9; 2.3	−5.94
Aloe-emodin	TP53	SER-227; GLU-221	2.2,2.8,2.4; 2.2	−5.08
Aloe-emodin	ALB	GLU-465; THR-478	2.6; 1.9	−4.46
Aloe-emodin	ESR1	TRP-383; GLU-380	1.8; 2.1	−5.28
Aloe-emodin	TNF	EU-145; PHE-143; HIS-140	2.3; 2.5,2.2; 2.6,2.2	−4.33
Aloe-emodin	STAT3	GLY-541; GLB-543; LYS-548	1.9; 2.5; 2.3	−3.03
Aloe-emodin	CASP3	THR-77; LYS-224; ASP-228	2.5; 2.5; 2.2	−4.80
Aloe-emodin	EGFR	ASP-800; CYS-797; ARG-841; ASN-842	2.5; 1.8; 2.1; 1.9	−5.24
Aloe-emodin	SRC	ESR-36; GLU-37	2.3; 2.2	−5.14
UC8	AKT1	GLU-242	1.9	−4.54
R4F	TP53	ALA-159; SER-227	1.9; 1.8	−4.48
MYR	ALB	GLN-417; LYS-534; TYR-497	2.4; 2.6; 2.1	−2.47
VQI	ESR1	ASP-369; ALA-307; LEU-308	2.1; 2.0; 1.8	−5.07
SO4	TNF	-	-	−5.43
KQV	STAT3	-	-	0.56
161	CASP3	ARG-101; LYS-105; ASP-146; ARG-149	1.7; 1.9; 3.0; 2.1	−2.72
03P	EGFR	ASP-984; GLN-812	2.1; 2.1	−3.43
821	SRC	THR-106	2.2	−5.43

#### 3.1.5 External validation of core targets

##### 3.1.5.1 mRNA, protein expression levels and survival analysis of core targets

**FIGURE 9:**
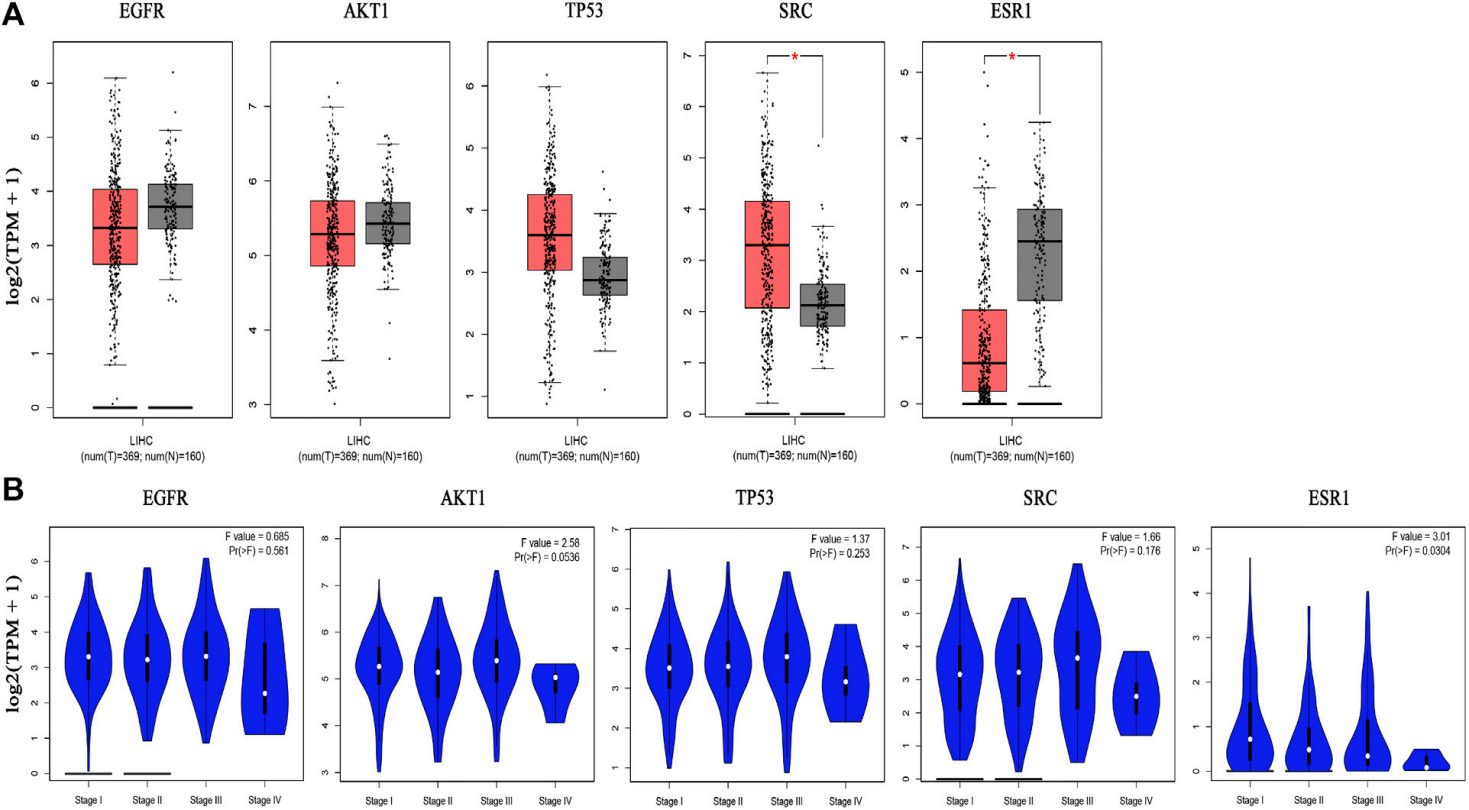
Hub gene expression in the GEPIA database. [**(A)** Box plot of hub gene mRNA expression levels in the GEPIA database. Red represents tumor tissues and gray represents normal tissues. Through the images we can find that SRC is highly expressed in hepatocellular carcinoma tissues compared with normal liver tissues and ESR1 is lowly expressed in hepatocellular carcinoma tissues compared with normal liver tissues. **(B)** Stage diagram of hub gene mRNA expression levels and pathological stages in the GEPIA database. There is a statistically significant difference of the expression level of ESR1 with its pathological stages (*p < 0.05*)].

**FIGURE 10:**
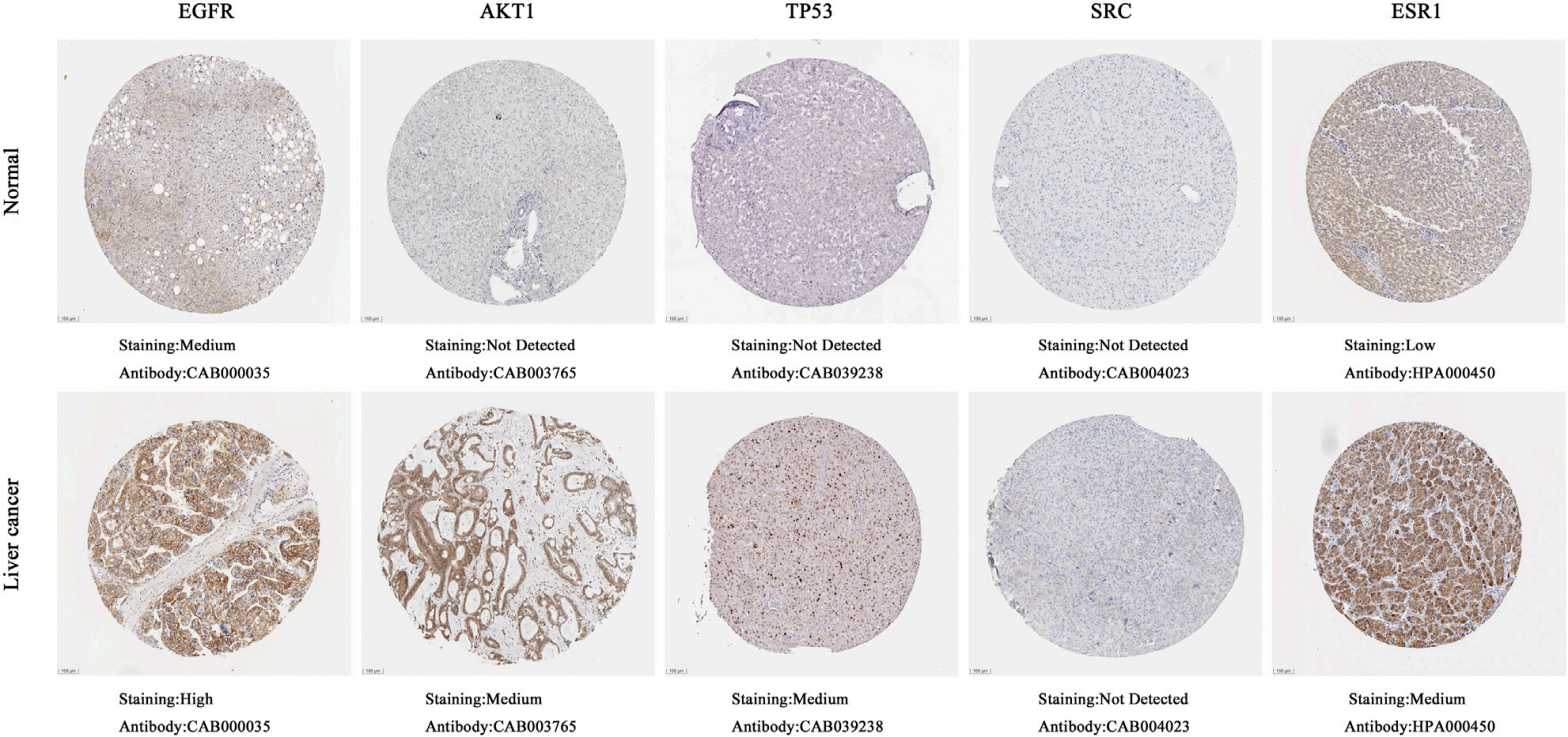
Immunohistochemical images of hub gene protein expression levels in the HPA database. (100 μm on the lower left quarter means all images were magnified 40 times under a microscope. The protein levels of EGFR, AKT1, TP53, and ESR1 were higher in hepatocellular carcinoma tissues than in normal liver tissues).

**FIGURE 11:**
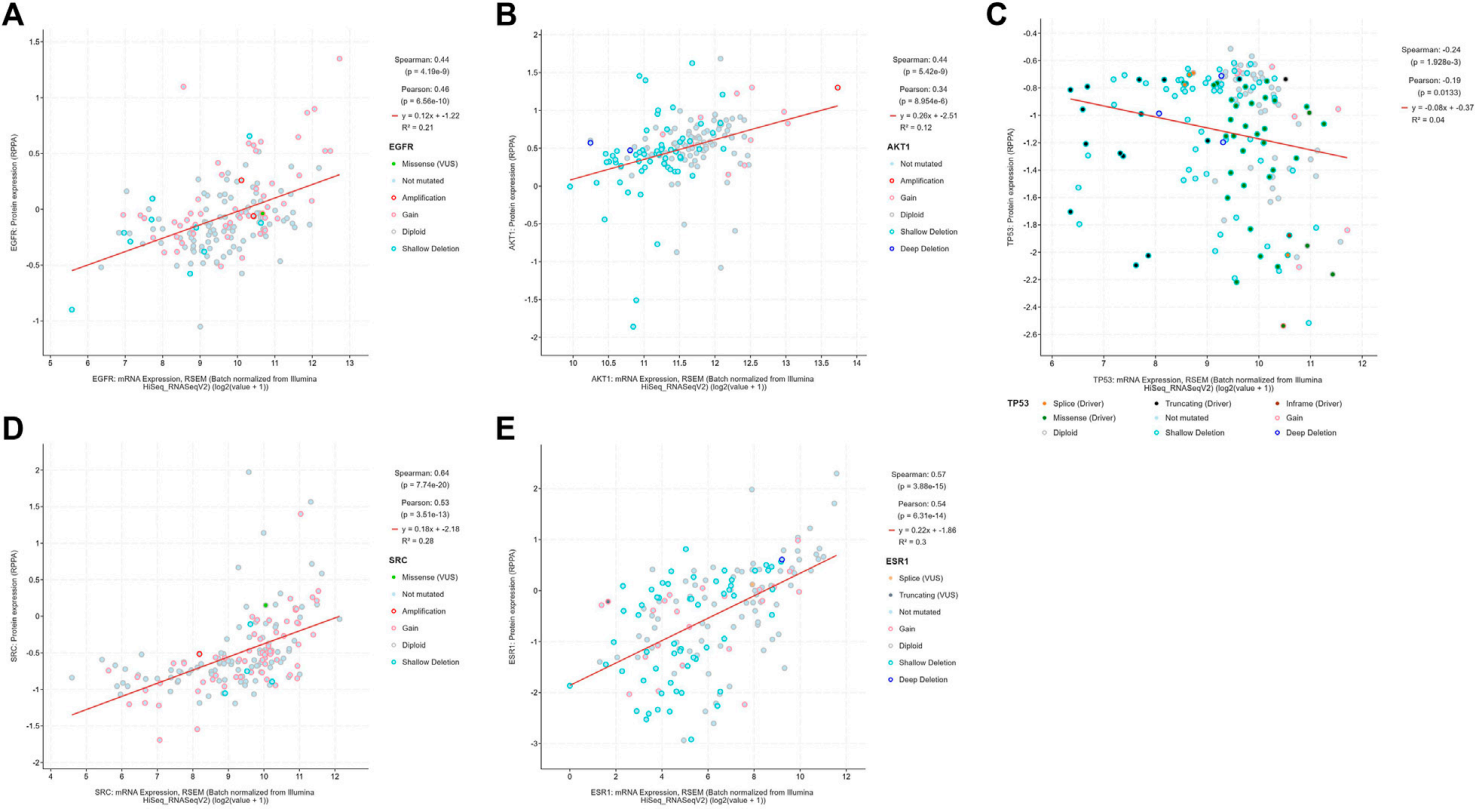
The diagram shows the correlation between the mRNA and protein levels of **(A)** EGFR, **(B)** AKT1, **(C)** TP53, **(D)** SRC, **(E)** ESR1 in hepatocellular carcinoma. (From the figure, it can be seen that the mRNA expression of EGFR, AKT1, SRC, and ESR1 were expressed consistently with the protein expression, while the mRNA expression of TP53 were expressed inversely with the protein expression).

**FIGURE 12:**
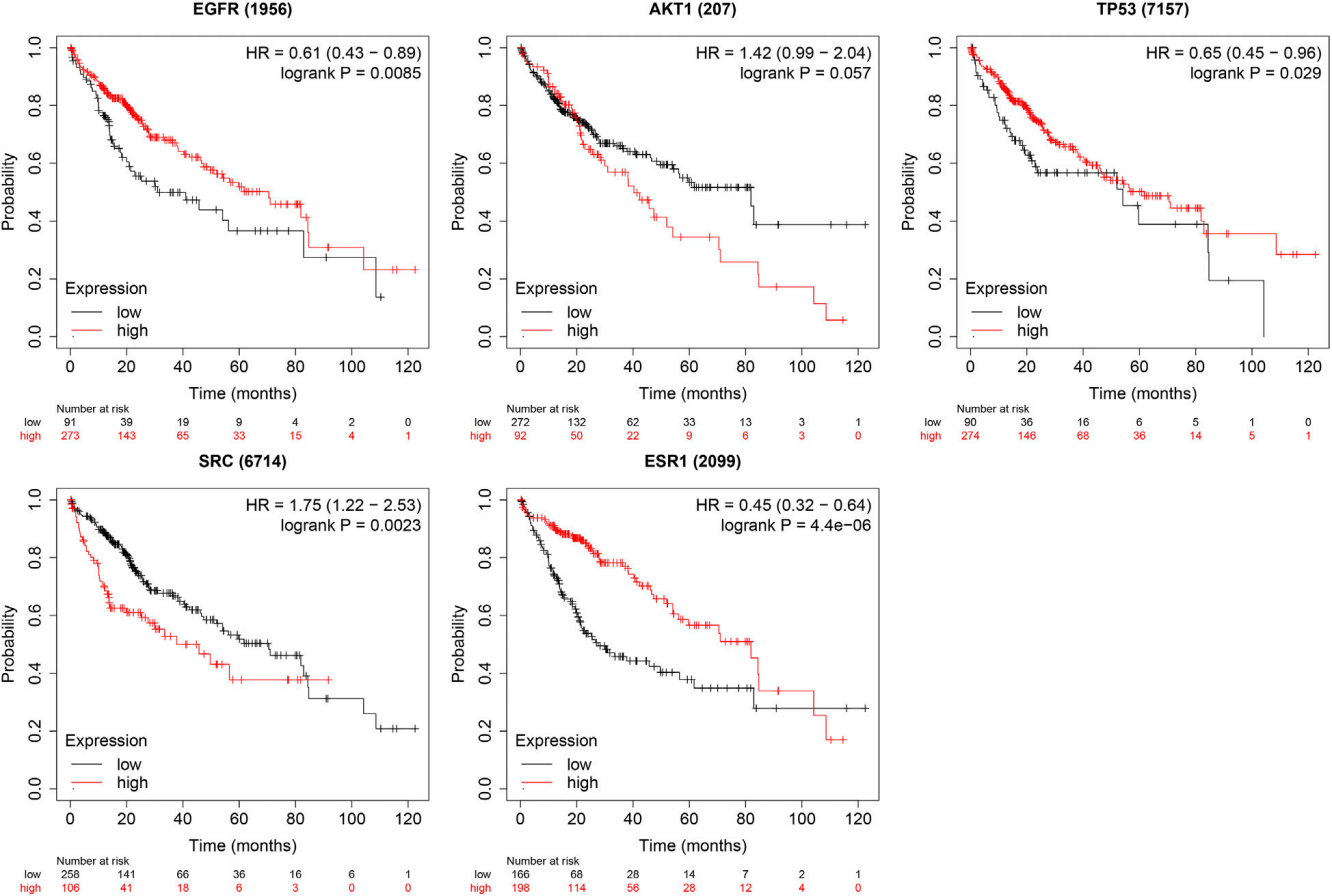
Relationship between high and low expression of core targets and survival prognosis in patients with hepatocellular carcinoma. [High expression of TP53 and ESR1 is beneficial to the survival of liver cancer patients, while high expression of SRC is harmful to patients (*p* < 0.05)].

The boxplot revealed the expression of five core targets in HCC compared with normal hepatocytes. The mRNA expression of ESR1 was lower in HCC cells *versus* normal hepatocytes; in contrast, the mRNA levels of SRC were higher in HCC cells (*p* < 0.05). In addition, we also performed a correlation analysis of the mRNA expression of core targets with the progression of HCC. We found that the expression of ESR1 was markedly changed with the development of HCC (Figure 9). Immunohistochemical staining images in the HPA database were used to determine the protein expression of core targets. We found elevated expression levels of ESR1, TP53, SRC, and EGFR. However, the expression profile regarding SRC proteomics was not discovered (Figure 10). Meanwhile, we found that the mRNA and protein expression levels of EGFR, AKT1, SRC, and ESR1 were positively correlated, while those of TP53 had a negative correlation (Figure 11). The curve obtained from the Kaplan-Meier plotter reflected the survival prognosis of HCC patients with high and low expression of the core targets. Patients with high expression of EGFR, ESR1, and TP53 were linked to a better prognosis *versus* those with low expression. This finding indicated that high expression of these three genes was a beneficial factor for the survival of patients with HCC. Patients with high expression of SRC had poorer prognosis than those with low expression. This evidence suggested that high expression of SRC was a detrimental factor for patients with HCC (Figure 12).

##### 3.1.5.2 Immune cell infiltration and genetic alteration of core targets

The relationship between core targets and immune cell infiltration in HCC was analyzed. CD4^+^ T cells, macrophages, neutrophils, and dendritic cells showed a positive correlation, whereas CD8^+^ T cells, B cells, and purity showed a negative correlation with the expression of EGFR in HCC. The abnormal activation of PI3K was positively associated with neutrophils and purity, while dysregulation of PI3K was negatively associated with immune infiltration of CD4^+^ T cells, CD8^+^ T cells, B cells, macrophages, and dendritic cells. ESR1 expression was positively correlated with B-cell infiltration and negatively correlated with the remaining five types of immune cells. ESR1 and TP53 were positively correlated with these six types of immune cells (Supplementary Figure S1A). Then, the mutation sites of the core genes in hepatocellular carcinoma were demonstrated (Supplementary Figure S1B). Mutation ratio and mutation types of core targets were further recorded. Of the 348 patients with HCC, 270 (60%) presented with mutations in core targets (Supplementary Figure S1C).

### 3.2 Experimental validation

#### 3.2.1 AE inhibited hepatic cancer cell growth *in vitro*

**FIGURE 13:**
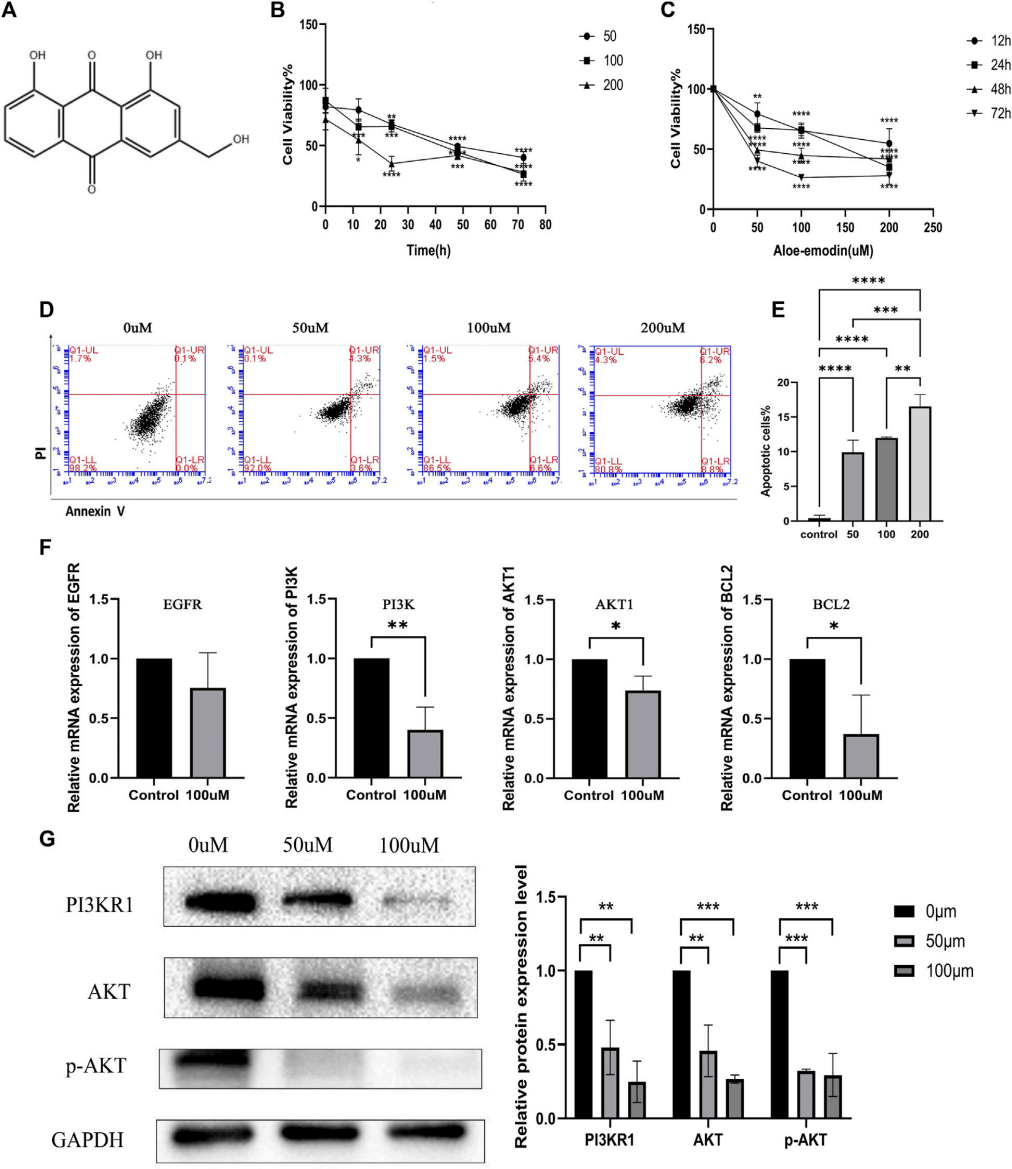
Inhibition of hepatocellular carcinoma cell proliferation, promotion of apoptosis, and inhibition of PI3K-AKT pathway by aloe-emodin. [**(A)** Chemical structure of Aloe-emodin. **(B)** Aloe-emodin inhibits HepG2 proliferation in a time-dependent manner. **(C)** Aloe-emodin inhibits HepG2 proliferation in a concentration-dependent manner. **(D)** Aloe-emodin promotes HepG2 apoptosis. **(E)** Proportion of apoptotic cells under different concentration treatments. **(F)** Aloe-emodin inhibits PI3K-AKT pathway at transcription expression level. **(G)** Aloe-emodin inhibits PI3K-AKT pathway at protein expression level (*p* < 0.05)].

The chemical structure of AE is presented in Figure13A. Cell viability was evaluated using the CCK8 assay to assess the anticancer effects of AE on HepG2 cells. The results revealed that AE inhibited the growth of HepG2 cells. Cell viability tended to decrease with the increasing duration of treatment and concentration of AE (Figures 13B,C). These data confirmed the antiproliferative effect of AE (*p <* 0.05).

#### 3.2.2 AE induced apoptosis of HCC cells

Annexin V-FITC and PI were designed to detect apoptosis in the early and late stages respectively. Cells in the lower right corner of the cross door were early apoptotic cells stained by FITC. Those in the upper right corner of the cross door were late apoptotic cells stained by PI (Figure 13D). The proportions of early and late apoptotic HCC cells increased in a concentration-dependent manner (*p* < 0.05) (Figure 13E).

#### 3.2.3 AE inhibited the mRNA expression of the PI3K-AKT signaling pathway

The sequences of primers used in this study are shown in Table 6. AE had a significant inhibitory effect on *EGFR*, *PI3K*, *AKT1*, and B-cell lymphoma 2 (*BCL2*) (Figure 13F). Abnormal activation of PI3K-AKT signal transduction in HCC is an important factor that inhibits apoptosis and promotes the growth and proliferation of tumor cells. This result demonstrated that AE could induce apoptosis in HCC cells via the PI3K-AKT signaling pathway (*p <* 0.05).

**TABLE 6:** Basic information of primer sequences.

Oligo name	Sequence (5′ to 3′)	Length	%GC	TM
EGFR-F	AGG​CAC​GAG​TAA​CAA​GCT​CAC	21	52.4	57.6
EGFR-R	ATG​AGG​ACA​TAA​CCA​GCC​ACC	21	52.4	57.2
PIK3R1-F	TGG​ACG​GCG​AAG​TAA​AGC​ATT	21	47.6	57.0
PIK3R1-R	AGT​GTG​ACA​TTG​AGG​GAG​TCG	21	52.4	56.9
AKT1-F	GTC​ATC​GAA​CGC​ACC​TTC​CAT	21	52.4	57.9
AKT1-R	AGC​TTC​AGG​TAC​TCA​AAC​TCG​T	22	45.5	55.6
BCL2-F	GGT​GGG​GTC​ATG​TGT​GTG​G	19	63.2	59.5
BCL2-R	CGG​TTC​AGG​TAC​TCA​GTC​ATC​C	22	54.5	57.4
GAPDH-F	GGA​GCG​AGA​TCC​CTC​CAA​AAT	21	52.4	57.2
GAPDH-R	GGC​TGT​TGT​CAT​ACT​TCT​CAT​GG	23	47.8	55.7

#### 3.2.4 AE inhibited the protein expression of the PI3K-AKT signaling pathway

Western blot was carried out to further confirm the role of the PI3K-AKT pathway in the treatment of hepatocellular carcinoma with aloe-emodin. The protein expression level of PI3KR1, AKT and phospho-AKT were attenuated by AE intervention compared with HepG2 cancer cells without AE intervention (*p* < 0.05) (Figure 13G). Together with qPCR results, our findings clearly illustrated the inhibitory effects of AE on HCC through PI3K-AKT signaling pathway.

## 4 Discussion

HCC is the most common type of liver cancer and associated with an extremely high mortality rate (Villanueva, 2019). The management of HCC can be divided into two aspects, namely, drug therapy and operative treatment. Nevertheless, after a long period of clinical application, these two treatments have been dwarfed, especially for patients with advanced liver cancer, so new anti-liver cancer methods need to be urgently investigated. AE, a natural anthraquinone derivative, has exhibited powerful antitumor effect against various types of cancer (e.g., lung, gastric, colon, and liver). Our research focused on the molecular mechanism underlying the effects of AE. And network pharmacology has been applied to elucidate potential mechanism involved in the treatment of HCC by AE. *In vitro* experiments were also utilized to confirm the therapeutic effect and mechanism of aloe-emodin.

First, we identified 63 overlapped targets presented in genes related to AE and HCC. These genes might be the key to the therapeutic effects of AE on HCC. GO analysis results of these 63 intersecting genes showed that AE may exert its therapeutic effect on HCC via negative regulation of the apoptotic process, response to drug, and positive regulation of the apoptotic process. Previous studies have illuminated the pro-apoptotic effect of aloe-emodin on HeLa cells (Li X. et al., 2022). It appears that apoptosis happens to be the main pathway through which AE affects HCC. The CCK8 assay and flow cytometry experiments further demonstrated that AE could inhibit the proliferation and promote the apoptosis of HepG2 cells in a concentration- and time-dependent manner. Numerous signaling pathways can be involved in the development of disease and drug action by acting on different genetic targets. Some signaling pathways detected through the KEGG analysis were related to inflammation and infection, which were consistent with previous evidence on AE. The pathways involved in cancer mainly included the PI3K-AKT signaling pathway, TNF signaling pathway, TP53 signaling pathway, and apoptosis. Hyperactivation of PI3K-AKT is present in various cancer types and regulates a broad spectrum of cellular mechanisms, including growth, apoptosis, proliferation and cycle (Fruman and Rommel, 2014). Mutations of multiple gene receptors in the cell membrane can activate the PI3K-AKT signaling pathway. PI3K catalyzes the conversion of PIP2 into PIP3, thereby phosphorylating the AKT serine/threonine kinase and promoting activation of the PI3K-AKT signaling pathway. Downstream members of the AKT pathway are associated with the cell cycle, apoptosis, survival, proliferation, glucose metabolism, etc (Khan et al., 2013). Dysregulation of the PI3K-AKT signaling pathway is common in patients with HCC (Sun et al., 2021). Our *in vitro* experiments directly demonstrated that AE could its inhibitory effects on HCC by downregulation of PI3KR1, AKT1, p-AKT. The TNF signaling pathway includes two types of receptors (i.e., TNFR1 and TNFR2) that play different roles by binding to TNF. TNFR1 binds to TNF to form a complex that can directly recruit CASP8 and initiate a protease cascade reaction. In addition, the receptor-interacting protein (RIP) in this complex induces the activation of NF-κB, which plays an important role in cell survival, proliferation, inflammation, and immune regulation (Chen and Goeddel, 2002). Binding of TNFR2 to TNF generates a completely opposite effect compared with that of TNFR1. TNFR2 significantly promotes cell migration and proliferation (Yang et al., 2018). The tumor suppressor TP53 can be activated in response to endogenous or exogenous stimulation (Kruiswijk et al., 2015). Activation of TP53 is closely connected with cell cycle arrest, metabolism, apoptosis, autophagy, senescence, etc. (Levine, 1997). Cancer patients with TP53 mutations are less sensitive to anti-tumor therapy and have a poorer prognosis *versus* those without mutations (Olivier et al., 2010). The apoptosis signaling pathway has an indispensable relationship with the occurrence of HCC. The activation of various signaling pathways ultimately plays a role in cell death or survival by influencing the apoptotic phenotype.

The top nine core targets (i.e., AKT1, TP53, ALB, ESR1, TNF, STAT3, CASP3, EGFR, SRC) were selected by protein-protein network. Proteins do not function as single substances, but as whole groups in biological processes (Zhao and Jiao, 2022). Protein interactions play a crucial role in biological signaling transduction and the transmission of biological information is the essence for cells to perform their functions (Mazandu et al., 2021) This signaling information is instrumental for us to study the mechanisms of disease (Moustakas and Heldin, 2009). Through PPI we were able to predict that these proteins with higher interaction strengths were more likely to be the targets of aloe-emodin therapeutic effect on hepatocellular carcinoma. Next, we conducted molecular docking to further validate the interaction strength between aloe-emodin and these nine proteins. Molecular docking results further revealed that AKT1, TP53, ESR1, EGFR, and SRC had energy <−5 kcal/Mol and more intense or similar binding energy than their original ligands. The data suggested that these five genes might be therapeutic targets. TP53 (p53) is a tumor suppressor protein. Under stressful conditions, p53 tightly regulates cell growth by promoting apoptosis and DNA repair. When cancer occurs, the p53 protein is mutated and loses its regulatory capacity (Luo et al., 2017). Previous studies have confirmed that AE triggers the accumulation of TP53, thereby inducing apoptosis in HepG2 cells (Kuo et al., 2002). Our survival analysis showed that hepatocellular carcinoma patients with concomitant p53 gene activation have a higher likelihood of survival. The discovery of the SRC gene arose from research on a chicken tumor virus called Rous sarcoma virus (Martin, 2001). SRC is a member of the tyrosine protein kinase family, which can regulate the apoptosis, proliferation, migration, invasion, and metastasis of tumor cells (Kim et al., 2009; Zhang et al., 2011; Dai and Siemann, 2019). Further research studies revealed that SRC was highly expressed in HCC (Yang et al., 2021), which was consistent with our result of SRC mRNA expression level in liver cancer. ESR1 has been established as a promoter of cancer in breast and the female reproductive system. Several clinical investigations have substantiated that mutation of ESR1 was associated with metastasis in breast cancer (Fuqua et al., 2014). Nevertheless, ESR1 can be a protective factor in HCC, and the progression of liver cancer is inhibited by ESR1 signaling *in vivo* (O'Brien et al., 2021). Patients with high ESR1 expression has higher survival rate compared with low ESR1 expression according to our results.

Our study confirmed the inhibitory effects of AE on HCC and the aberrant activation of PI3K-AKT was also affirmed in our *in vitro* experiments. Through the decreased expression of PI3KR1, AKT1, and BCL2 mRNA levels compared with control group, we demonstrated that the inhibition of hepatocellular carcinoma by aloe-emodin could be mediated through the PI3K-AKT signaling pathway. The downregulation of PI3KR1, AKT and phospho-AKT in protein levels further reinforced our findings. And through network pharmacology, we also verified the involvement of AE in other signaling pathways for the treatment of HCC. Meanwhile, our article has some limitations. Our utilization of the database is incomplete to the extent which leads to reduced credibility of the selected genes. The signaling downstream of the PI3K-AKT signaling pathway need to be further explored. Still and all, our research affirms the therapeutic role of aloe-emodin on hepatocellular carcinoma through the regulation of PI3K-AKT signaling pathway.

## 5 Conclusion

This study elucidated the regulatory mechanism of AE in the treatment of HCC through network pharmacology and cell experiments. The results demonstrated that multiple signaling pathways are involved in this process. AE could exert its therapeutic effect by binding to EGFR, SRC, AKT1, TP53, and ESR1. Our findings also verified the importance of the PI3K-AKT signaling pathway through which AE might exert its therapeutic effect on HCC. However, the regulatory effects of AE on signaling targets downstream of the PI3K-AKT pathway remain unknown, and more scientific practice needs to be engaged to improve the understanding of the therapeutic effects of AE. In conclusion, our study provides a more solid theoretical basis for the clinical application and exploration of the mechanism of AE in the treatment of HCC.

## Data Availability

The original contributions presented in the study are included in the article/Supplementary Material, further inquiries can be directed to the corresponding author.
