# Supplementary material for: Exploring the mechanism of aloe-emodin in the treatment of liver cancer through network pharmacology and cell experiments
Source: Front Pharmacol. 2023 Oct 12;14:1238841. doi: 10.3389/fphar.2023.1238841 (PMC10600456; doi:10.3389/fphar.2023.1238841)
Supplement: Supplementary file 1 [file DataSheet1.doc]

**Supplementary Figure 1.** Immune cell infiltration and genetic alteration of hub targets. (A. Immune cell infiltration of core targets. B. Core targets mutation sites in HCC. C. Data shows that 209 of 348 patients (60%) has genetic mutations in these targets, and mutation types are identified.)
